# A tug-of-war to control plant emission of an airborne alarm signal

**DOI:** 10.1007/s44154-023-00135-9

**Published:** 2023-11-17

**Authors:** Jie Hao, Junfei Ma, Hua Shi, Ying Wang

**Affiliations:** 1 Plant Pathology Department, University of Florida, Gainesville, 32611 USA; 2 State Key Laboratory for Conservation and Utilization of Bio-Resources in Yunnan, Yunnan Agricultural University, Kunming, 650201 China

**Keywords:** Salicylic acid, Methyl-salicylate, NAC2, SAMT1, SABP2, Airborne defense

## Abstract

Aphids represent a major threat to crops. Hundreds of different viruses are aphid-borne. Upon aphid attack, plants release volatile organic compounds (VOCs) as airborne alarm signals to turn on the airborne defense (AD) of neighboring plants, thereby repelling aphids as well as reducing aphid fitness and virus transmission. This phenomenon provides a critical community-wide plant protection to fend off aphids, but the underlying molecular basis remains undetermined for a long time. In a recent article, Gong et al. established the *NAC2*-*SAMT1* module as the core component regulating the emission of methyl-salicylate (MeSA), a major component of VOCs in aphid-attacked plants. Furthermore, they showed that SABP2 protein is critical for the perception of volatile MeSA signal by converting MeSA to Salicylic Acid (SA), which is the cue to elicit AD against aphids at the community level. Moreover, they showed that multiple viruses use a conserved glycine residue in the ATP-dependent helicase domain in viral proteins to shuttle NAC2 from the nucleus to the cytoplasm for degradation, leading to the attenuation of MeSA emission and AD. These findings illuminate the functional roles of key regulators in the complex MeSA-mediated airborne defense process and a counter-defense mechanism used by viruses, which has profound significance in advancing the knowledge of plant-pathogen interactions as well as providing potential targets for gene editing-based crop breeding.

This brief article highlights the recent results of Gong et al. (2023), who have provided critical insights into two main aspects: 1) the methyl-salicylate (MeSA)-mediated airborne defense (AD) against aphids and viruses, as well as, 2) the cooperation between aphids and viruses in countering the MeSA-mediated AD. While plants are unable to move from place to place after germination, they are resilient in adaption to the ever-changing environment and respond to various biotic and abiotic stresses for survival. Insects, including aphids, represent a major threat to crops, not only because insect bites are directly destructive to plants but also due to the spread of almost all types of plant pathogens including bacteria, fungi, protozoa, nematodes, viruses, etc. (Purcell and Almeida 2004). Aphid attack activates plant immunity, including salicylic acid (SA)-based immunity that integrates defense at the whole-organism level through systemic acquired resistance (SAR) (Vlot et al. 2009; Foyer et al. 2015) and the emission of volatile compounds to mediate AD in neighboring plants (Shulaev et al. 1997).

SA, a well-known phytohormone in plant immunity, is rapidly accumulated upon pathogen challenge through isochorismate- or phenylalanine-based biosynthesis (Ngou et al. 2022). A fraction of SA is converted to MeSA by the salicylic acid-carboxylmethyltransferase-1 (SAMT1) at infection sites (Ding and Ding 2020). MeSA is subsequently transported to distant part of plants through the phloem as a mobile signal (Park et al. 2007). This systemic transportation of the SA signaling is critical for SAR, a survival-promoting immune strategy of plants (Zhang and Dong 2022). Reversibly, MeSA can be converted back to SA by a methylesterase (salicylic acid-binding protein-2, SABP2) to activate SAR upon pathogen and insect attack (Forouhar et al. 2005). Therefore, with SA as a central component, SAMT1 and SABP2 contribute to SAR by regulating the homeostasis between SA and MeSA (Vlot et al. 2021).

When aphids attack plants, plants generate and emit volatile organic compounds (VOCs) to activate AD in neighboring plants (Pickett and Khan 2016). A pioneer work identified methyl jasmonate as the first airborne VOC functioning in AD (Farmer and Ryan 1990). However, despite that the composition of VOCs is complex and variable depending on the plant species and the types of pathogen attack, MeSA is the major component of VOCs against phloem-feeding aphids (Gong et al. 2023). A previous study reported that MeSA mediates AD via being converted back to SA in the model system of tobacco plants infected with tobacco mosaic virus (Shulaev et al. 1997). Nevertheless, the detailed molecular basis of MeSA-mediated AD remains elusive.

The Liu Laboratory at Tsinghua University (Beijing, China) and their collaborators have been working on the plant defense mechanism underlying plant-virus interactions for decades. Their recent work illustrated NAC2 from *Nicotiana benthamiana* as an interactor with the 1a protein of cucumber mosaic virus (CMV) through immuno-pulldown followed by mass spectrometry analysis. This interaction is pivotal for CMV infection as the viral titers increased in the *nac2* knockout plants generated by using the CRISPR-Cas9 technology (Gong et al. 2023). Interestingly, a fortuitous observation revealed that more green peach aphids colonized the *nac2* knockout plants as compared with the wildtype (WT) plants (Gong et al. 2023), despite *N. benthamiana* being known as a non-preferred host of green peach aphids (Honglin and Georg 2023). Further characterization showed that *NAC2* mediates AD to repel aphids in neighboring plants through MeSA biosynthesis.

Gong and colleagues then employed RNA-Seq analysis and found that *SAMT1* expression was consistently reduced in the *nac2* knockout plants. Chromatin immunoprecipitation followed by qPCR, yeast-one-hybrid assay, and electrophoretic mobility shift assay all showed that NAC2 binds to the promoter of *SAMT1*. Luciferase reporter imaging assay using *SAMT1* promoter showed that NAC2 acts on the *SAMT1* promoter to promote transcription in cells. Knowing that SAMT1 is the key enzyme in methylating SA to MeSA, these data well support the notion that the *NAC2*-*SAMT1* module regulates the production of MeSA.

One key question is whether the *NAC2*-*SAMT1* module responses to the volatile MeSA signaling. To tackle this question, Gong and colleagues found the supporting evidence that *NAC2* and *SAMT1* expressions were induced after the airborne treatment of volatized MeSA and the exogenous application of SA. Interestingly, the same treatment cannot induce either *NAC2* or *SAMT1* expression in the transgenic *N. benthamiana* plants expressing bacterial *NahG*, which encodes the salicylate hydroxylase known to degrade SA. This supports that volatized MeSA is likely converted to SA to activate the *NAC2*-*SAMT1* module. Importantly, the accumulation of MeSA but not the SA production was inhibited in the *nac2* knockout plants after aphid attack, confirming that the *NAC2*-*SAMT1* module specifically regulates the MeSA production.

A critical link between the volatized MeSA signal and the activation of the *NAC2*-*SAMT1* module is the enzyme that converts MeSA to SA. SABP2, though not considered as a specific SA receptor, has long been known as the key enzyme regulating the conversion of MeSA to SA (Yan and Dong 2014). Gong et al. confirmed that the physiological concentration of MeSA (3 nM) was able to compete with SA for binding with SABP2. The *sabp2* loss-of-function mutant failed to respond to the volatile MeSA treatment for SA production or aphid repellence, indicating that *SABP2* is critical for perception of the MeSA signal. Furthermore, the volatized MeSA showed no difference between WT and the *sabp2* plants after external application of SA, indicating that *SABP2* is not involved in MeSA emission.

More than 40% plant viruses can be transmitted by aphids to infect a plethora of crops (Gong et al. 2023). CMV deploys its 2b protein to inhibit the plant jasmonate signaling thereby attracting aphids for transmission (Wu et al. 2017). Interestingly, although either CMV infection or attack by virus-free aphids can induce MeSA production in plants, plants attacked by CMV-carrying aphids emitted less MeSA as compared with emission from plants attacked by virus-free aphids (Gong et al. 2023). Moreover, plants attacked by CMV-carrying aphids failed to initiate effective AD to prevent aphid attack and consequently the viral transmission in neighboring plants. To comprehend these observations, Gong et al. further explored the link between the NAC2-CMV1a interaction and the *NAC2*-*SAMT1* module. Multiple experiments together demonstrated that CMV1a interacts with NAC2 and shuttles the transcription factor to the cytoplasm, where NAC2 is subject to 26S proteasome-based protein degradation. The ATP-dependent helicase domain (HD) of CMV1a was confirmed to mediate the interaction with NAC2 by using the luciferase complementation imaging assay, and the glycine at the 983 position (G983) in the HD of CMV1a was predicted as the residue with the nearest physical proximity to NAC2 by using the AlphaFold-Multimer program. The CMV1a protein with a G983D mutation failed to alter NAC2 subcellular localization or suppress NAC2-mediated activation of SAMT1 expression. Transgenic plants expressing the 1a^G983D^ mutant failed to repress either MeSA volatilization or plant repellant to aphids. All these lines of evidence support that CMV1a modulates the *NAC2*-*SAMT1* module through direct interaction with NAC2 (Fig. 1).

**Fig. 1:**
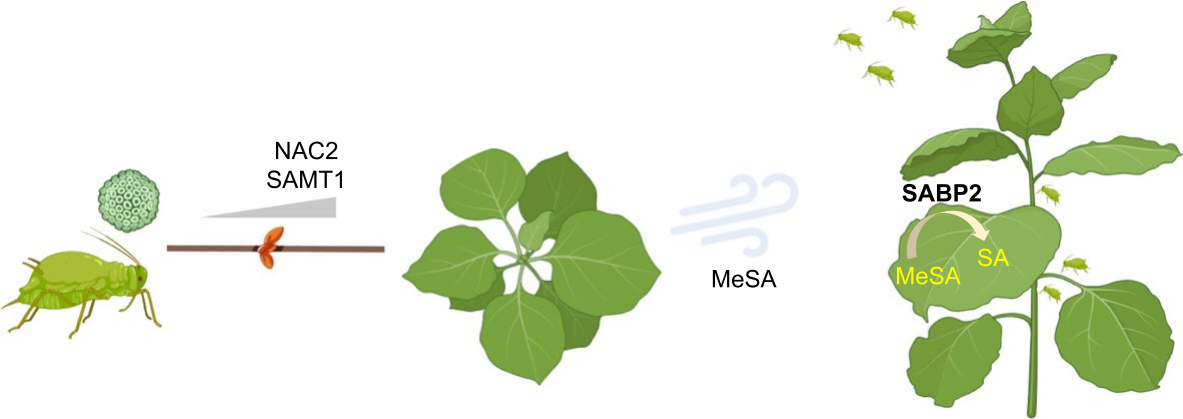
A model illustrating the tug-of-war between plants and virus-carrying aphids in controlling AD. Aphids and viruses synergistically attenuate the expression of the NAC2-SAMT1 module, which is critical for emission of volatized methyl-salicylate (MeSA) to activate airborne defense (AD) in neighboring plants. In neighboring plants, MeSA is converted to SA by SABP2 to activate immune responses. AD is pivotal to expel aphids in neighboring plants. Images from Biorender were used for generating this figure

Gong and colleagues also explored other aphid-borne viruses, such as potato virus Y (PVY) from the genus Potyvirus. Like CMV, PVY infection was enhanced in the *nac2* plants and affected volatized MeSA emission after aphid attack. AD was confirmed to reduce PVY transmission. WT plants, which were adjacent to plants either mock damaged without aphid or attacked by PVY-carrying aphids, showed no difference in attracting PVY-carrying aphids or PVY transmission, supporting that aphid and PVY teamed up to inhibit AD in neighboring plants. Interestingly, the HD domain found in CMV is widely existing in viral proteins encoded by many aphid-borne viruses, including PVY. The glycine residue at the 347 position of PVY CI protein is equivalent to the G983 of CMV1a. The WT PVY-CI, but not the PVY-CI^G347D^, was able to shuttle NAC2 to the cytoplasm for degradation. Therefore, a critical glycine residue in the HD domain in viral proteins likely represents a conserved mechanism in modulating the *NAC2*-*SAMT1* module to mitigate the MeSA-mediated AD and promote aphid-transmission of viruses, benefiting aphids and viruses but harming crops.

Aphid-borne viruses pose a worldwide threat to nowadays agriculture (Jeger et al. 2023). Pesticides control alone cannot meet the desired expectation to eliminate aphids in fields and often cause other problems such as environment pollution, reducing beneficial insects including pollinators, residual chemicals on produce, etc. Breeding new crop varieties takes long time, which may not meet the pressing need to increase crop yields to feed a growing global population. In this sense, Gong et al. provided very critical insights into the molecular basis of AD and the counter-defense mechanism by aphid-borne viruses. This thorough study explicates the functional roles of many components in the complex AD process and provides potential targets for future gene editing-based crop breeding. In addition, this study can stimulate future investigations to broaden the knowledge of SA-based immunity (e.g., the role of NAC2 in SAR), how viruses lacking the HD or the conserved glycine residue overcome AD, SA-mediated immunity and AD in plant-aphid-virus tripartite interactions, etc.

## Data Availability

Not applicable.

## Abbreviations

VOC: Volatile organic compound
AD: Airborne defense
MeSA: Methyl-salicylate
SA: Salicylic Acid
SAR: Systemic acquired resistance
SAMT1: Salicylic acid-carboxylmethyltransferase-1
SABP2: Salicylic acid-binding protein-2
CMV: Cucumber mosaic virus
WT: Wildtype
PVY: Potato virus Y
HD: ATP-dependent helicase domain.
